# Role of pause duration in primary progressive aphasia

**DOI:** 10.1080/02687038.2024.2366285

**Published:** 2024-06-21

**Authors:** Roelant Ossewaarde, Yolande Pijnenburg, Antoinette Keulen, Roel Jonkers, Stefan Leijnen

**Affiliations:** a Center for Language and Cognition, Research School for Behavioral and Cognitive Neurosciences, University of Groningen, Groningen, The Netherlands; b Institute for ICT, HU University of Applied Science, Utrecht, The Netherlands; c Alzheimercentrum UMC Amsterdam, Neurology, Amsterdam, The Netherlands

**Keywords:** Aphasia, primary progressive (MeSH ID D018888), primary progressive nonfluent aphasia (MeSH ID D057178), frontotemporal dementia (MeSH ID D057180), speech disorders (MeSH ID D013064), automatic fluency assessment

## Abstract

**Aims:**

To detect differences in speech fluency in separate primary progressive aphasia syndromes (PPA) using automated analysis techniques. The resulting linguistic features are evaluated for their use in a predictive model to identify common patterns in speakers with PPA. As fluency is observable in audio recordings, its quantification may provide a low-cost instrument that augments spontaneous speech analyses in clinical practice.

**Methods and Procedures:**

Speech was recorded in 14 controls, 7 nonfluent variant (nfvPPA) and 8 semantic variant (svPPA) speakers. The recordings were annotated for speech and non-speech with Kaldi, a common toolkit for speech processing software. Variables relating to fluency (pause rate, number of pauses, length of pauses) were analyzed.

**Outcomes and Results:**

The best fitting distribution of pause duration was a combination of two Gaussian distributions, corresponding with pause categories *short* vs. *long*.

Group level differences were found in the rate of pauses and proportion of silence: nfvPPA speakers use more short pauses relative to long pauses than control speakers, and the duration of short and long pauses is longer; svPPA speakers use more longer pauses relative to short pauses. Their short pauses are significantly shorter than those from control speakers.

Participants in both PPA groups pause more frequently. SvPPA speakers are typically perceived as fluent. However, our analysis shows their fluency patterns to be distinct from control speakers, if the long-short distinction is observed.

**Conclusions:**

Automatic measurements of pause duration show meaningful distinctions across the groups and might provide future aid in clinical assessment.

## Introduction

Individuals with dementia often experience a decline of their ability to use language. Language problems have been reported in dementia caused by Alzheimer’s disease, Lewy Body dementia or frontotemporal dementia. The term Primary Progressive Aphasia is used to describe a neurodegenerative condition in which the primary, dominant symptom is a progressive language disorder.

Three variants of Primary Progressive Aphasia are commonly acknowledged, after Gorno-Tempini et al. (2011): the *non-fluent* variant of Primary Progressive Aphasia (nfvPPA) is characterized by agrammatism and/or slow or labored speech, the *semantic* variant (svPPA) by fluent but increasingly empty speech with affected naming and word comprehension, and the *logopenic* variant (lvPPA), which is aphasia with anomia, poor repetition and relatively spared word comprehension. The first two variants are most often associated with frontotemporal lobar degeneration (FTLD), whereas the logopenic variant is commonly associated with Alzheimer’s disease pathology (Rohrer et al., 2012).

Although most clinical PPA presentations fit the current framework, heterogeneous and mixed phenotypical manifestations may challenge the formation of clear linguistic criteria for recognizing the disorder (Ingram et al., 2020) or its subcategories (Bergeron et al., 2018; Leyton et al., 2015; Louwersheimer et al., 2016).

Biomarkers are “medical signs – that is, objective indications of medical state observed from outside the patient – which can be measured accurately and reproducibly” (Strimbu & Tavel, 2010). According to the published criteria of Gorno-Tempini et al. (2011), a diagnosis of PPA requires confirmation of specific speech and language symptoms. As the diagnostic process includes the assessment of the impairment of language, measurements of language variables may be considered a biomarker.

The measurement of the degree of language impairment usually involves aphasia battery tests, such as the Boston Diagnostic Aphasia Examination (Goodglass et al., 2001), Western Aphasia Battery (Kertesz, 2006), Aachen Aphasia Test (Miller et al., 2000), Psycholinguistic Assessment of Language Processing in Aphasia (Kay et al., 1996) and Comprehensive Aphasia Test (CAT; Swinburn et al., 2004). In particular the CAT has been adapted to a wide range of languages (Fyndanis et al., 2017). Subtyping of PPA is usually performed with tests that measure the production of connected speech, the speech motor abilities, the ability to repeat words and sentences, the ability to name objects or actions, auditory comprehension and the degree to which reading and writing is affected (Europa et al., 2020).

Connected speech is one of the most valuable of these domains (Matias-Guiu et al., 2022). It is important for diagnostics (Boschi et al., 2017), and the ability to produce connected speech in spontaneous language highly correlates with the perceived quality of life of persons with aphasia (Cruice et al., 2003). The tasks to elicit connected speech range from carrying out a simple conversation to describing a given picture or retelling a story. Such tests are easy to administer and

relatively non-invasive for test takers. A single sample of a person’s language contains many different variable types that are usable for profiling the language production abilities.

The amount of data and level of sophistication required for the analysis of linguistic variables depends on their type (Ossewaarde et al., 2020). Acoustic variables can be observed even in short amounts of speech and require no analysis of linguistic content. Their usefulness for predictive disease models has been shown for several neurodegenerative diseases (amyotrophic lateral sclerosis, ALS, Simmatis et al., 2024; Alzheimer’s Disease, Parlak et al., 2023; Parkinson’s Disease, Holmes et al., 2000).

Although elicitation of connected speech is relatively easy, its transcription is laborious. Software can help reduce the associated costs. There have been several attempts to develop a pipeline of automated analysis for dementia analysis (e.g., Le et al., 2018; Liu et al., 2023; Qin et al., 2018) and to identify which features can be measured with a high degree of certainty (e.g., Faroqi-Shah et al., 2020; Haider et al., 2020; Luz et al., 2021; Mahajan & Baths, 2021; Sadeghian et al., 2021; Themistocleous et al., 2021).

In the context of PPA, fluency is the most important acoustic variable to distinguish the nonfluent variant from the other variants. Fluency is defined as continuity, smoothness, rate, and effort in speech production, with a *fluency disorder* defined as the interruption of the flow of speech characterized by atypical rate, rhythm or by disfluencies (American Speech-Language-Hearing Association, 1993). The accepted view is that fluency patterns in spontaneous speech are affected only in persons with nfvPPA, not in persons with svPPA (Gorno-Tempini et al., 2011).

Lack of fluency leads to pauses in speech. A pause is defined as an interval where the probability of acoustic energy produced by the speaker falls below a certain threshold. From a functional perspective, some sounds (e.g., *uh* or *uhm*) are produced as placeholders of pauses, leading to a distinction between filled and unfilled pauses. In this study we focus on the automatic analysis of unfilled pauses in relation to the different types of PPA. A divergent ratio of speech and pauses is typically perceived as speech with affected fluency (Cucchiarini et al., 2002; Kormos & Dénes, 2004).

Studies that use ensemble-analyses of the language of persons with dementia have found time and rate of both phonation and pauses to be affected, in persons diagnosed with MCI (Roark et al., 2011) or with PPA (Fraser et al., 2014; Fraser et al., 2013). Jarrold et al. (2014) reported shorter pause durations in fluent PPA patients than in controls; pause-to-word ratio is one of the variables in the model of Pakhomov et al. (2010) that discriminates between the fluent and nonfluent PPA-variants and behavioral variant FTD (Geraudie et al., 2021). In other domains, such as Alzheimer’s Disease detection, the inclusion of pause and disfluency information improved classification models significantly, e.g., Nasreen et al. (2021) and Yuan et al. (2021).

Labeling fluency requires labeling of speech and non-speech events on a temporal scale. Manual alignment of temporal events is hard and expensive. Automatic annotation, however, is readily available because detecting whether someone is speaking (’Voice Activation Detection’) is a well-studied problem in the realm of speech processing, with implementations with acceptable performance for most purposes.

Determining the fluency profile of a speech fragment can be a diagnostic tool to recognize disorders that influence fluency. It can also aid in quantifying the effects of the disease, which is relevant due to the heterogeneity of the linguistic profiles of persons with PPA. Dis- or nonfluency is well detectable by humans because it is audibly present in casual speech – however, an explicit profile that includes quantitative data is required before a comparison can be made between participants, or of the same participant at different moments in time.

A large-scale study (5.5 hours of speech in five languages) on silent pauses in conversational and read speech (Campione & Véronis, 2002) suggests that the distribution of pauses of healthy speakers is best approximated with a multimodal distribution with three centers: brief (*<* 200 ms), medium (200–1000 ms) and long (*>* 1000 ms) pauses. Their comparison of three pause studies of French indicates thresholds between short and medium length pauses at 300 ms, 200 ms, and 180 *−* 250 ms.

For English, Goldman-Eisler (1968) suggested a two-way distinction (long-short), with a threshold at 250 ms. This value was confirmed by Kirsner et al. (2003), which used a data driven approach to derive a bimodal distribution with cutoff at about 255 ms, however with large individual variation. Fraser et al. (2013) separated two categories, short pauses (200–400 ms) and long pauses (*>* 400 ms). For Dutch, a similar (non-individualized) threshold of about 250 ms was found (De Jong & Bosker, 2013). A study of Greek (Angelopoulou et al., 2018) found the threshold to be higher, at 339 ms.

By definition, individuals with the nonfluent PPA variant are expected to show longer and more frequent pauses than non-brain damaged individuals. Given the increased effort in language production that all individuals with PPA face, a distinction between speakers with PPA and control speakers is expected as well.

In this study, we set out to investigate how PPA speakers can be classified using fully automated measurements of their fluency through application of a specific type of speech processing (Voice Activation Detection, VAD) to spontaneous speech of persons in three groups: control, nfvPPA and svPPA speakers. The resulting fluency features are used in a predictive model to identify common fluency patterns. It is well-known that pauses are different in patients and controls and between patients with nfvPPA and svPPA. Our study contributes a precise and objective quantification, yet reliable and efficient, of the fluency patterns.

## Materials and methods

Language samples were collected from two groups of Dutch speaking participants: one control (*n* = 14) group and one group of persons with PPA (*n* = 15), under the care of neurologists at the Alzheimer Center (Amsterdam UMC). They were selected for inclusion in this study based on a neurologist’s assessment of probable PPA according to the diagnostic criteria of Gorno-Tempini et al. (2011). Participants in the patient group are part of the Amsterdam Dementia Cohort (Van der Flier et al., 2014), which means that they undergo a standardized healthcare pathway as part of their clinical workup that includes a battery of diagnostic tests. In 12 cases amyloid biomarker assessment had taken place.

Participants in the control group were selected for this study from a cohort of volunteers in brain research studies (Dutch Brain Research Registry; Zwan et al., 2021). They were matched demographically by the selection algorithm of the registry. Control participants were included when they were native speakers of Dutch and had no history of neurological or psychiatric disorders, as determined by a questionnaire. Demographic characteristics are reported in Table 1. Clinical assessment outcomes for participants in the patient groups are reported in Table 2.

**Table 1.** Main clinical and demographic characteristics.

Variable	Control	nfvPPA	svPPA
Number of participants	14	7	8
Number of language samples	14	12	16
Women (%)	57	50	69
Months since symptom onset	n/a	41.42	34.57
Age at language recording	62.5 *±* 7.9	66.8 *±* 6.3	66.1 *±* 3.0
MMSE	n/a	26.00 *±* 1.73	27.50 *±* 0.55

Data are shown as mean ± standard deviation or frequency (%). Kruskal-Wallis test indicated no statistically significant different distributions with *alpha <*= 0.05. nfvPPA: nonfluent variant of PPA, svPPA: semantic variant of PPA.

**Table 2.** Clinical assessment of PPA participants.

	nfvPPA		svPPA	
	Mean	SD	Mean	SD
**Global measures**				
MMSE	26.0	1.73	27.5	0.55
CDR	0.3	0.3	0.5	0.0
GDS^b^	1.0	1.0	1.8	1.0
FAB^c^	15.0	2.4	17.5	0.6
**Attention**				
Digit Span Forward	5.0	0.8	5.5	1.2
Digit Span Backward	4.0	0.8	4.8	0.4
Extended Digit Span Forward	11.2	1.5	12.3	2.8
Extended Digit Span Backward	7.2	1.3	9.7	0.8
Letter Digit Subsitution Test	42.2	15.8	52.5	8.2
**Episodic memory**				
15WT, after 5 trials	38.7	1.24	40.0	2.2
15WT recall	7.8	0.5	8.2	3.9
15WT false negatives	1.0	0.8	0.3	0.5
15WT false positives	0.8	0.5	1.2	0.4
CFT Recall	11.8	3.9	18.5	3.0
**Language and semantic memory**				
Animal Fluency Test	19.5	4.4	11.8	3.1
VAT A naming	12.0	0.0	8.0	3.3
Letter Fluency Test A	4.5	1.9	6.3	2.3
Letter Fluency Test D	9.2	2.9	10.3	2.0
Letter Fluency Test T	7.2	3.9	10.0	2.7
CFT copy	34.8	2.5	35.2	1.5

MMSE = Mini-Mental State Examination (Kok & Verhey, 2002); CDR = Clinical Dementia Rating (Morris, 1993); GDS = Geriatric Depression Scale (Yesavage, 1988); FAB = Frontal Assessment Battery (Dubois et al., 2000); 15WT = 15 Word Test, Dutch version of Rey Auditory Verbal Learning Test (Van den Burg et al., 1985); CFT = Rey Complex Figure Test; VAT = Visual Association Test (Lindeboom & Schmand, 2014); DAT Letter Fluency Task (Schmand et al., 2008).

For each participant, discourses on three different topics (relating to events that took place in the past, present and future) were elicited. In addition, a conversation based on the picture description of the CAT-NL (Swinburn et al., 2004) was recorded. Characteristics of the speech fragments are reported in Table 3. If possible, the elicitation of the three topics was repeated with patients at follow-up visits after at least 3 months.

**Table 3.** Variables observed in the spoken description of the picture.

	Control Group		Nonfluent Variant		Semantic Variant		ANOVA			
Variable	Mean	SD	Mean	SD	Mean	SD	F	DF	p	Contrasts
Phonation time (s)	**75.07**	**31.54**	**44.07**	**19.97**	66.05	23.67	4.91	2.38	0.013	control*>*nfvPPA
Locution time (s)	97.53	45.06	119.75	44.62	137.74	55.79	2.46	2.38	0.099	*no constrasts*
Pause rate^a^	**0.23**	**0.09**	**0.40**	**0.08**	**0.41**	**0.12**	16.07	2.38	<0.001	control*<*nfvPPA, control*<*svPPA
Proportion of silence^b^	**0.21**	**0.11**	**0.61**	**0.16**	**0.50**	**0.13**	33.95	2.38	<0.001	control*<*nfvPPA, control*<*svPPA

^a^The average number of pauses per second.

^b^The proportion of silence relative to the locution time.

In some cases, individuals contributed multiple times, albeit at different visits to the clinic. Due to the heterogeneous disease development, it was assumed that each sample was effectively an independent data point. To confirm, a linear mixed-effects model was fitted (lme4 for R, v 4.3.2; Bates et al., 2015) with participant identity as random effect. The intraclass correlation coefficient (ICC1; Bliese, 2000) was used to quantify the influence of the random factor, as a measure of the relative independence of data points given a clustering of data points where elicitations are from the same person (Bosker & Snijders, 2011).

Elicitation from participants in the control group was done using the Google Meet videoconferencing tools due to social distancing measures that were in place at the time of data collection. Elicitation from participants in the nfvPPA and svPPA groups was done in-person during clinic visits. Sound was recorded using a TASCAM DR-40X recorder. The recorder has two mounted microphones which were turned towards the interviewer and interviewee. In addition, both the interviewer and the interviewee wore a head mounted microphone (Shure WH20 XLR).

The audio was first labeled for the identity of the speaker (’speaker diarization’), using X-Vectors (Kaldi; Snyder et al., 2017). An additional variable was introduced in the decision layer that codes which of the four microphones was spoken into, which further informs the diarization decision.

Segments of the interviewer of detectable length were removed by training a speech recognizer (built with Kaldi v5.5; Povey et al., 2011) on the specific voice properties of the interviewer.

Audio recordings were then analyzed for the length and duration of pauses using a Voice Activation Detection (VAD) filter. Experimentation with three different algorithms to decide Voice Activation^1^ indicated no meaningful performance differences. For this study, the Kaldi algorithm was used, combined with a decision procedure that labels audio parts as either speech or non-speech.

**Figure 1.**
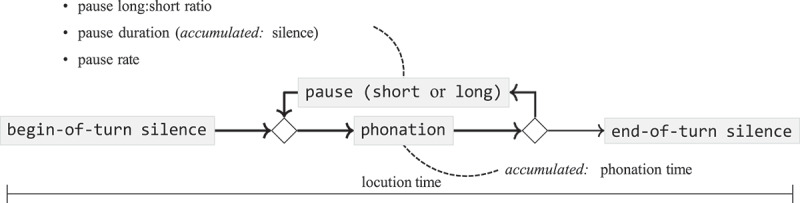
The variables in this study related to the elements of the narration.

The measures derived after applying the VAD filter are (cf. Figure 1):

1. Locution time: The duration of the description (in s).^2^
2. Phonation time: The cumulative duration of speech segments in the description.
3. Pause rate: The number of pauses relative to a unit of time. *accumulated*: phonation time.
4. Proportion of silence: The cumulative duration of pauses relative to locution time.

A pause is a continuous segment labeled as non-speech with a duration of at least 50 ms (Baken & Orlikoff, 2000). After analysis of the distribution of pauses, the *number of pauses* (relative to time: *pause rate*) was divided into two categories, *number of short pauses* and *number of long pauses*. The *amount of silence* was operationalized as the cumulative duration of pauses in a fragment. The *length of pauses* is derived as a function of the amount of silence and the number of pauses in a fragment (cf. Eq. 1).

(1) $$\mathrm{average}\;\mathrm{length}\;\mathrm{of}\;\mathrm{pauses}=\frac{\mathrm{amount}\;\mathrm{of}\;\mathrm{silence}}{\mathrm{number}\;\mathrm{of}\;\mathrm{pauses}}$$

The following statistical analyses were performed with R^3^

Between-group differences were studied with analysis of variance for continuous data (ANOVA). The motivation is to test the null hypothesis that the speech speakers with PPA have identical characteristics with regards to their phonation time, locution time, pause rate and proportion of silence. Overall between-group differences were further analyzed with Tukey post-hoc pairwise comparisons, with Bonferroni correction applied where applicable. Because we are working with small sample sizes, we confirmed the ANOVA outcomes with a permutational multivariate analysis of variance (PERMANOVA; adonis2 test as implemented in the R vegan package). We used the Bray-Curtis distance metric which does not assume normality.

The distribution of pauses is often log-normal (Campione & Véronis, 2002): their distribution is heavily skewed towards shorter pauses. We transformed the duration data to a log_10_ scale and binned them in intervals of equal length. If the durations of pauses randomly vary around some central measurement, their distribution will shape as a Gaussian curve, due to the Central Limit Theory (Frank, 2009). If there are multiple types of pauses, the final distribution will shape as the addition of multiple Gaussians, each with their own mean and standard deviation. Hence, we account for the distinction between short and long pauses by assuming that the data is multimodal in nature. The generating distribution is assumed to be the sum of multiple distinct Gaussians. The parameters of each Gaussian were estimated using the mixtools-package (Benaglia et al., 2009) in R. The parameters of the distributions are compared using standard t-tests.^4^

To understand the relationship between the three variables that feature in Equation 1, a Bayesian probability model was trained to predict the number of pauses as a function of silence and length of pauses, and their interaction.

The advantage of the Bayesian method is that it can use all of the data to construct a model, retaining all its uncertainty properties, independent of any generating distribution. This facilitates comparisons between variables best modeled using varying distributions such as the normal or log-normal. The Bayesian analysis allows inferences about parameters to be drawn from the posterior distribution of a trained model. The inferences are valuable even if the input data is as strongly biased as ours.

The motivation is that this allows estimation of the generating distribution function for the number of pauses, and thus a measure to report on the differences between participant groups. The joint valuation of silence and number of pauses yields a measure that can be used to predict average pause length (cf. Equation 1).

The model is a Bayesian multinomial GLM with R and trained to recognize what kind of speaker generated a fragment of audio, where participant kind is equated with either control group or the diagnosis of any of the studied diseases.

The automated analyses provided the raw data on which we ran the Bayesian analysis. The audio was divided in 20-second fragments and processed through the VAD filters. The Bayesian estimation was performed with Stan (Stan Development Team, 2023) through the rethinking interface (McElreath, 2023) using a version of Markov chain Monte Carlo (MCMC).

The data is count data (rate) with varying means and variances, cf. Table 4. We hypothesize that due to the analytical nature of the data, its observed skew, and the Gaussian fits when log-transformed, the *Gamma-Poisson* (negative binomial) probability density function best describes the generating distribution of the data. This probability density function is similar to the binomial/Poisson distribution, with a shape parameter (mu), however with an extra parameter to control scaling its variance (beta).

**Table 4.** Characteristics of component pause distributions.

Group	Short Pauses			Long Pauses		
	Mean	SD	Lambda^a^	Mean	SD	Lambda
Control	165.22	86.78	0.29	759.97	505.31	0.71
nfvPPA	285.20	253.26	0.37	1691.74	151.78	0.63
svPPA	71.62	17.96	0.07	767.49	794.64	0.93

^a^Lambda = the mixing ratio of the two Gaussians into the mixed model.

Mixed-effect models were used because they provide a better fit compared to repeated measures ANOVA: mixed-effect models allow one to take into account the speaker’s variability by computing random slopes and intercepts for each participant, thus taking into effect the individual speaker’s differences.

## Results

The Kruskal-Wallis test of the age of the participants at recording did not indicate a significant difference between the ages in the control, svPPA and nfvPPA groups: H(2) = 1.97, *p* = 0.37.

Table 3 presents the characteristics of the fragments and the comparisons of the observed fluency variables between the control, nfPPA and svPPA groups.

A Generalized Linear Effects model with a random slope for subjects serves as an indicator for the bias introduced because some participants contributed multiple samples. After model fitting, ICC estimates, as calculated based on a two-way mixed effects model design with multiple measurements, indicate absolute agreement as 0.42. Values below 0.5 usually indicate poor reliability, hence relative independence of the samples.

ANOVA testing showed significant effects (*α* = 0.05) of at least two of the three participant groups on *phonation time* (F(2, 38) = [4.91], *p* = 0.013), *pause rate* (F(2, 38) = [16.07], *p <* 0.001) and on the *proportion of silence* (F(2, 38) = [33.95], *p <* 0.001). PERMANOVA tests indicated similar pseudo-F scores and identical *p*-values.

Tukey’s HSD Test for multiple comparisons found that the mean value of *phonation time* was significantly different between the control and nfvPPA groups (*p <* 0.05, 95% C.I. = [61.5, 88.63]), *pause rate* was significantly different between the control and nfvPPA groups (*p <* 0.001, 95% C.I. = [0.18, 0.28]) and between the control and svPPA groups (*p <* 0.001, 95% C.I. = [0.34, 0.46]), and that the mean value of *proportion of silence* was significantly different between the control and the nfvPPA groups (*p <* 0.001, 95% C.I. = [0.14, 0.28]) and between the control and the svPPA groups (*p <* 0.001, 95% C.I. = [0.54, 0.69]).

There were significant effects of participant group on each of the variables: participants in both patient groups produced pauses at a higher rate and spoke with relatively more silence. Participants with the nonfluent variant generated shorter narratives than participants in the control group, as measured both by the number of words and by the time they spoke. Participants with the semantic variant produced more words, but did not speak longer.

**Figure 2.**
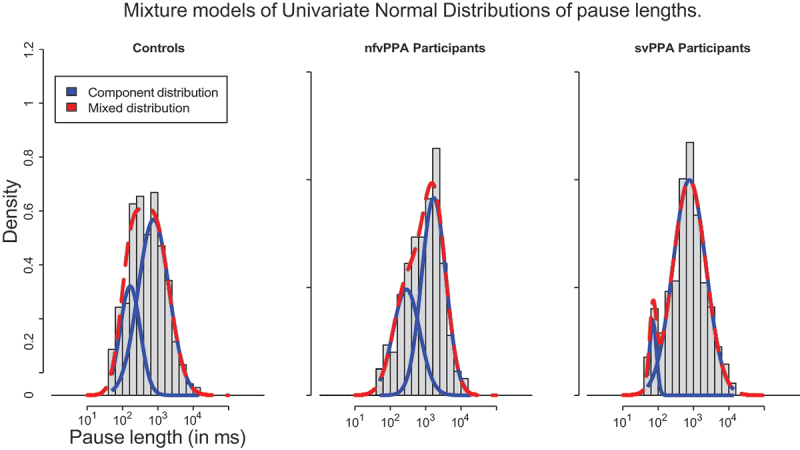
The distribution of pauses > 50 ms, with overlaid a mix of two normal distributions that best describe the data. The cutoff between long and short pauses is assumed to be the point where the first component Gaussian meets the second component Gaussian.

The distributions of pauses of each of the three main groups are plotted in Figure 2. Visual inspection of the histograms, with their estimated normal distributions overlayed, shows that a mix of two Gaussians – and not three, as was found by Campione and Véronis (2002) — provides a good fit to the data. The parameters of the Gaussian curves are reported in Table 4.

Comparison of the parameters allows the formulation of fluency pattern blueprints. Significance tests were applied to random samples from the contributing Gaussians (*n* = 1*e*3). Compared to controls, nonfluent variant PPA participants make *longer* short pauses (*t*(1251.66) = *−*14.80, *p < *.001, *d* = *−*0.66) and *longer* long pauses (*t*(1187.64) = *−*58.01, *p < *.001, *d* = *−*2.59). Comparison of the two lambda values shows that the ratio of short:long pauses is different, with nfvPPA participants generating more short pauses when compared to controls.

SvPPA participants make, unlike nfvPPA participants, *shorter* short pauses (*t*(1251.66) = *−*14.80, *p < *.001, *d* = *−*0.66). There is no significant difference with regards to the longer pause category. Comparison of the two lambda values shows that for the svPPA group, there are relatively more longer pauses in comparison to controls and nfvPPA participants.

**Figure 3.**
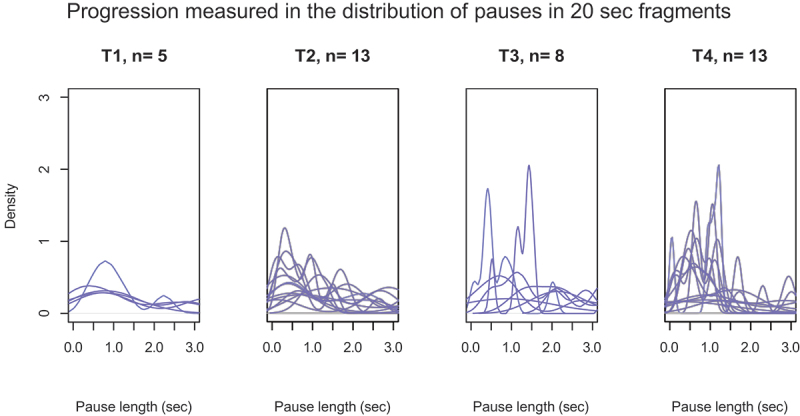
Density plots for participant 11 (svPPA) show the distribution of pauses in 20 sec fragments at different moments of elicitation.

Conversations were held at up to four different moments during the progression of the disease. Analyzing within the patient group, there was no significant effect of moment of speech on any of the variables. However, on the individual level, a change was visible between moments for some patients. An example is plotted of data from a person with svPPA in Figure 3, which shows an increase in the number of shorter pauses in later moments.

## Bayesian analysis of number of pauses

**Figure 4.**
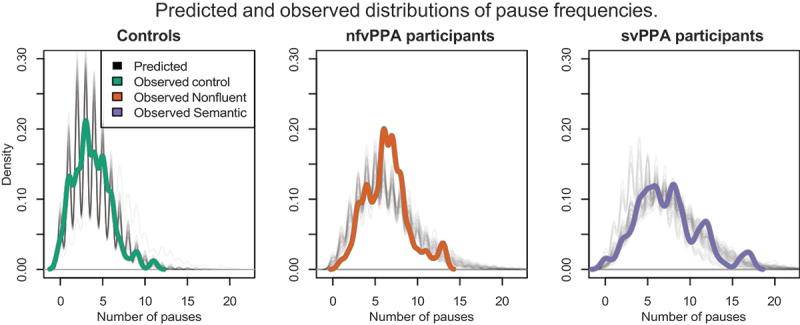
Density plots of observed data (colored lines) compared to density plots of the inferred Gamma-Poisson distribution of number of pauses (solid lines) show the fit of the inferred model with the actual data.

As posterior predictive check for the Bayesian analysis of the data, the predicted and observed densities for the count of pauses per fragment is plotted in Figure 4. The visual overlap of the density lines yields support for the choice of the negative binomial as generating distribution for the number of pauses.

The MCMC chains for every parameter have converged and are long enough (Gelman-Rubin R^^^ 1.00, number of effective chain samples 3796, confirmed by visual inspection of the chain’s traceplots) to provide stable estimates.

**Figure 5.**
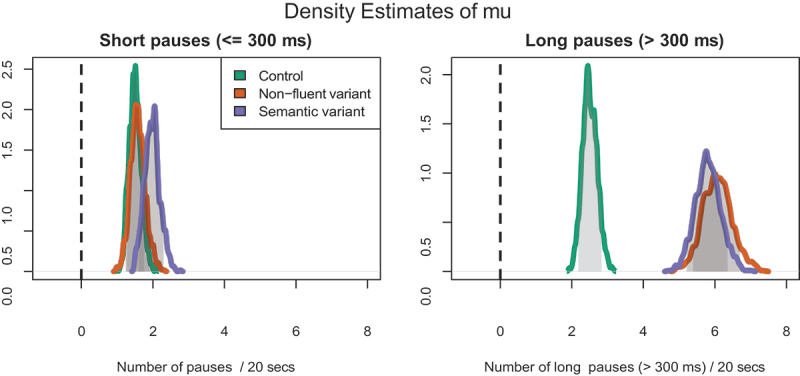
The distribution of plausible difference in pause rate between patients and controls measured as the distance between estimated mu-parameters of the different participant groups, split in long and short pauses. Values are compared from the Highest Density Intervals of each of the distributions (p = 0.89, shaded in gray). Overlapping HDI’s indicate a lack of support to reject null hypothesis.

In Bayesian analyses, the overlap of parameter values in the posterior serves as an indication of the probability that they are similar – if the overlap is less than a given threshold, the parameter values are considered to be different. In the posterior drawn from the model there is little overlap between the mu-parameter for language disturbed individuals and that for controls. The differences of the mu-parameters of the groups are visualized in Figure 5. The distributions do not include zero, which indicates a high probability that the populations are indeed different (Kruschke, 2014). The Bayesian analysis corresponds with the ANOVA analyses of the same data.

Figure 5 shows that the division between short and long pauses provides features can be used to distinguish groups. The cutoff value between short and long pause length is 293 ms. It is determined by the saddle point of the two component Gaussians from controls in Figure 2, i.e., where the probability density of the long pause Gaussian exceeds the density of the long pause Gaussian.

Between all three groups, there is overlap in the distribution of the mu-parameter for (the rate of) short pauses, which indicates that there is no support for the hypothesis that these groups are different with regards to this feature. The density estimates for the *rate of long pauses* do not overlap between the control group and either of the patient groups, which indicates support for the hypothesis that the nfvPPA and svPPA variant speakers use more long pauses than control group speakers.

The results of this study are summarized in Table 5.

**Table 5.** Summary of findings.

Variable		nfvPPA vs control	svPPA vs control	nfvPPA vs svPPA	Reported in
Phonation time		less	*n.d*.^*a*^	less	Table 3, phonation time
Amount of silence		more	more	*n.d.*	Table 3, proportion of silence
Pause long:short ratio		fewer long vs short	more long vs short	fewer long vs short	Table 4, lambda
Pause duration	Short pauses	longer	shorter	longer	Table 4, mean short pauses
Pause duration	Long pauses	longer	*n.d.*	longer	Table 4, mean long pauses
Pause rate	Overall	more pauses	more pauses	*n.d.*	Table 3, pause rate
Pause rate	Short pauses	*n.d.*	*n.d.*	*n.d.*	Figure 5
Pause rate	Long pauses	more	more	*n.d.*	Figure 5

^a^*n.d*. = no difference.

## Discussion

In this study, we set out to automatically quantify the fluency patterns of PPA speakers so that a machine learning algorithm can distinguish control speakers from those with either the nonfluent or the semantic variant of PPA. Our main findings were that after distinguishing between short and long pauses in a speech fragment, their rate and lengths provide a discriminative profile that distinguishes between control speakers and PPA speakers and also within the two groups of speakers with PPA, between individuals with the nfvPPA and the svPPA variant.

The data, both from controls and from PPA speakers, can be described by a combination of two Gaussians, each representing a separate pause class. Pause rate, in this study operationalized as the number of pauses relative to a set duration of time, is distributed according to a Gamma-Poisson distribution, which is a skewed alternative to the Poisson distribution.

We hypothesize that the two Gaussians are the result of two separate processes that generate interruptions. The longer pauses are perhaps generated for deliberate, cognitive reasons, such as indicating a linguistic cue or pause for thinking, in line with studies that identify longer pauses with distinct loci in the sentence or discourse. The shorter pauses are so short that they are perhaps best understood as interruptions for physical reasons such as breathing.

The observed distribution of pauses and its distinction into two types is relevant for the study of silent pauses: separation of types and exponential scaling of our data is required before their normal distributions shows, cf. Angelopoulou et al. (2018) and Campione and Véronis (2002). The linguistic meaning attributed to silence (e.g., Ephratt, 2008; Lestary et al., 2018; Scollon & Wong-Scollon, 2013) is usually associated with longer pauses – a separate category allows better cross-linguistic comparisons, and given pauses’ apparent communicative functions, specific attention in the case of neurodegenerative diseases that affect language or communication.

The literature shows variation in the typology of pauses and the ways in which they are measured. Our methodology involves an automated way of finding the separation point between short and long pauses. In our data, on Dutch, the cutoff appears at 293 ms (*±*0.61). This cutoff is higher than the value of 250 msthat is often used, however well below the

threshold of 339 ms that was reported for Greek in a similar task by Angelopoulou et al. (2018). Our data shows a clear bimodal distribution, whereas the studies for French discussed by Campione and Véronis (2002) found that a three-way distinction (short-medium-long) was best fitting. The cross-linguistic difference in distributions and threshold values is a future avenue of research.

Our results show that participants in both svPPA and nfvPPA groups pause more frequently, and that both groups generate overall more silence than control participants. For the nfvPPA group, this is conform expectations, because disfluencies are phenotypes of the nfvPPA diagnosis.

SvPPA speakers are typically perceived as fluent. However, the automated analysis applied to SvPPA speakers in our study shows their fluency pattern to be distinct from control speakers: their short pauses are shorter than those of controls, and they generate long pauses relatively more frequently (compared to short pauses). This distinct pattern is contrary to the common assumption that the fluency of persons with svPPA is unaffected.

The differing short-long pause patterns between svPPA and nfvPPA also support the argument that a separation between long and short pauses, previously advocated by Campione and Véronis (2002), is important when analyzing fluency. In this study, we show that that separation improves a predictive model that distinguishes between the non-fluent and semantic PPA variants.

A possible explanation of the distinct SvPPA pausing pattern is that svPPA speakers typically have word finding difficulties, which may cause the increased pause rate in this participant group.

In this study, the pauses are derived by software, without human labeling – this allows uniform quantification of pausing patterns, which in turn may allow better insight in threshold values for pathological speech. It is also efficient, because humans require significantly more time for temporal markup of speech. The volume of pauses that we were able to analyze allows for insight in the general distribution patterns of pauses and their parameters. The automated results in our study are comparable to results found after manual pause transcription (Campione & Véronis, 2002; Ferré, 2021; Potagas et al., 2022 *inter alia*).

The outcomes of this study have clear clinical relevance, as they show that distinguishing between different types of pauses in short fragments of spontaneous speech already gives clear cues for the distinction between controls and PPA speakers, and within the group of PPA speakers between those with the nonfluent and the semantic variant.

A yet unanswered question concerns the change of fluency within individuals during the progression of the disease. Informal inspection of the data shows no clear patterns across all participants – which is in line with the typical heterogeneous character of the disease. Further studies with larger cohort sizes may provide data with enough statistical power to address this issue.

The description of a situational picture, the stimulus of our study, is not as truly spontaneous as a real conversation (Prins & Bastiaanse, 2004; Shriberg, 2005). True conversations require timing and turn-taking, and may tax the cognitive system more due to the combination of comprehension and production that is inherent to multiparty discourse. For example, one case reports an agrammatic speaker with aphasia who produces speech with different characteristics when describing a given image than when engaging in normal conversation (Beeke et al., 2003). The analysis of truly spontaneous speech is perhaps more ecologically valid, as it is the majority of ordinary language use. An extension of our study would be the application of the fluency detection algorithms to determine the stability of our findings regarding pause lengths, their distributions and their ability to discriminate speakers.

One limitation of our study is that the elicitation in the control group was performed in a different setting (online) than the elicitation in the other groups (in-person, at the clinic). Interviews with control speakers were specifically planned, and they spoke from the comfort of their own homes. Interviews with participants were held right before or after their clinical consultation with a neurologist, in a hospital examination room. These are likely stress-increasing factors. Future research may study whether that leads to better or worse performance.

Automatic pause detection will err consistently for some sounds; for example, most VAD algorithms (including the one used in this study) suffer to recognize fricative consonants (Makowski & Hossa, 2020). This introduces a bias in the data. However, its systematic nature suggests that subsequent analysis is not unduly influenced by the bias.

Given that affected spontaneous speech is part of the diagnostic criteria of PPA and its subtypes, software that facilitates its analysis carries impact. Automatic speech analysis is faster, more consistent and does not need the presence of a specialist to interpret the data. It will therefore be helpful in the early detection of PPA, maybe as one of the first clues of a possible disorder.

As a suggestion for future research, it is likely that the temporal distribution of pauses, the locus of the nonfluency within a sentence, deviates in persons with word finding problems from healthy speakers. This will require more data because measurements will be made on the clausal level. Additionally, it will require a separate tier to indicate clause boundaries.

We conclude that measuring pauses using a Voice Activation Detection algorithm yields measurements that are informative enough to be predictive in a classification model. The fluency of svPPA or nfvPPA speakers is distinct from that of healthy control speakers.

## Notes

1. 1: a custom implementation of the algorithm of Ramírez et al. (2004); 2: the Matlab implementation of the algorithm of Drugman et al. (2015), 3: the VAD detection algorithm bundled with the WSJ recipe of Kaldi.

2. ^2^The locution time includes the turn-initial and turn-final silences (if any); these silences are not counted as pauses because they do not interrupt the phonated segments.

3. R version 4.1.2, with packages rethinking (McElreath, 2023), mc‐stan (Stan Development Team, 2016) and vegan (Oksanen et al., 2022).

4. As implemented in the stats-package of R (R Core Team, 2017).
